# Association between Air Pollution and Lipid Profiles

**DOI:** 10.3390/toxics11110894

**Published:** 2023-10-31

**Authors:** Yi Zhang, Jiaqi Shi, Ying Ma, Nairui Yu, Pai Zheng, Zhangjian Chen, Tiancheng Wang, Guang Jia

**Affiliations:** 1 Department of Occupational and Environmental Health Sciences, School of Public Health, Peking University, Beijing 100191, China; 1710306142@pku.edu.cn (Y.Z.); 1610306221@pku.edu.cn (J.S.); mayingmmyy@163.com (Y.M.); 1810306112@bjmu.edu.cn (N.Y.); zhengpai.mail@gmail.com (P.Z.); jiaguangjia@bjmu.edu.cn (G.J.); 2 Beijing Key Laboratory of Toxicological Research and Risk Assessment for Food Safety, School of Public Health, Peking University, Beijing 100083, China; 3 Department of Laboratory Medicine, Peking University Third Hospital, Beijing 100191, China; tcwang@bjmu.edu.cn

**Keywords:** air pollution, particulate matter, blood lipid, cardiovascular disease, dyslipidemia

## Abstract

Dyslipidemia is a critical factor in the development of atherosclerosis and consequent cardiovascular disease. Numerous pieces of evidence demonstrate the association between air pollution and abnormal blood lipids. Although the results of epidemiological studies on the link between air pollution and blood lipids are unsettled due to different research methods and conditions, most of them corroborate the harmful effects of air pollution on blood lipids. Mechanism studies have revealed that air pollution may affect blood lipids via oxidative stress, inflammation, insulin resistance, mitochondrial dysfunction, and hypothalamic hormone and epigenetic changes. Moreover, there is a risk of metabolic diseases associated with air pollution, including fatty liver disease, diabetes mellitus, and obesity, which are often accompanied by dyslipidemia. Therefore, it is biologically plausible that air pollution affects blood lipids. The overall evidence supports that air pollution has a deleterious effect on blood lipid health. However, further research into susceptibility, indoor air pollution, and gaseous pollutants is required, and the issue of assessing the effects of mixtures of air pollutants remains an obstacle for the future.

## 1. Introduction

Air pollution is an important public health problem endangering human health. The Global Burden of Disease, Injuries, and Risk Factors (GBD) study 2019 showed that over 6 million premature deaths were attributed to air pollution (3.75 million deaths of all male death and 2.92 million deaths of all female death) [1]. Both ambient particulate matter pollution and household air pollution were among the top 10 risk factors in terms of DALYs. According to the report of the World Health Organization in 2021, cardiovascular disease-related deaths accounted for about 60% of premature deaths caused by outdoor air pollution [2]. Dyslipidemia is an important cause of cardiovascular disease, as it can promote the formation of atherosclerosis and induce cardiovascular disease [3,4,5]. High LDL cholesterol, solely based on its impact on cardiovascular disease, ranked among the top ten risk factors for DALYs in 2019. It is found that air pollution is related to lipid metabolism dysfunction, which causes abnormal blood lipid levels [6,7,8]. Abnormal blood lipids may be one of the important intermediate links in the process of cardiovascular diseases caused by air pollution. Therefore, there is a need to discuss the relationship between air pollution and blood lipids and related health problems. This review briefly summarizes the epidemiological research on the associations between air pollution and abnormal blood lipids and the mechanisms of air pollutants affecting blood lipids, and further extends the understanding of the potential cardiovascular and cerebrovascular disease risk and metabolic dysfunction diseases related to blood lipid disorders caused by air pollution.

## 2. Air Pollution and Blood Lipids

### 2.1. Association between Air Pollution and Blood Lipids

Increasing epidemiological evidence shows that air pollution exposure is associated with adverse changes in blood lipid levels. A systematic review and meta-analysis of 22 studies in 2019 showed that the triglyceride (TG) level increased by 3.14% and 4.24% for every 10 μg/m^3^ increase in long-term exposure to particles with diameters ≤10 μm (PM_10_) and nitrogen dioxide (NO_2_), respectively [6]. However, no other associations between lipid indexes and air pollutants were significant. Due to the limited number of short-term exposure studies, only a meta-analysis of long-term exposure was conducted in Gaio’s review, and only three studies were included in the meta-analysis [6]. In the systematic review section, some included studies also reported significant associations between high-density lipoprotein cholesterol (HDL-C), low-density lipoprotein cholesterol (LDL-C), and total cholesterol (TC) and air pollution. However, the results of different studies varied greatly, and the differences in exposure time, exposure population, pollutant composition, and research methods limited the comparison of research results [6]. 

After 2019, there have been many new studies on the association between air pollutants and blood lipids. A recent meta-analysis [9] showed that long-term air pollution was associated with adverse changes in blood lipid levels. TC was positively correlated with long-term exposure to particles with diameters ≤1.0 μm (PM_1_), particles with diameters ≤2.5 μm (PM_2.5_), PM_10_, and carbon monoxide (CO); TG was positively correlated with long-term exposure to PM_10_; HDL-C was negatively correlated with long-term exposure to PM_1_, PM_10_, sulfur dioxide (SO_2_), and CO; and LDL-C was positively correlated with long-term exposure to PM_2.5_ and PM_10_. Because air pollution persists and its harm to people is long-term, most studies are aimed at long-term air pollution exposure [10,11,12,13,14,15,16,17,18,19,20,21,22,23,24,25,26,27]. A few studies have shown that short-term air pollution levels can also bring about changes in blood lipids [28,29,30,31,32,33,34,35]. The role of short-term air pollution is helpful for explaining the mechanism and process of blood lipid changes caused by air pollution. In addition, the meta-analysis of Li et al. showed that with the increase in lipid variability (especially TC and HDL-C variability), the mortality risk of cardiovascular disease and all-cause mortality also increased [36]. If short-term air pollution exposure will affect blood lipid levels, then huge fluctuations in short-term air pollution, such as sandstorms, may affect blood lipid variability.

Epidemiological research exploring the relationship between air pollution and lipid levels has incorporated repeated-measures, cross-sectional, and time-series analysis [28,34,37,38]. Repeated measurements are frequently utilized in panel studies involving small sample sizes for assessing short-tomedium-term air pollution exposures, as it is crucial to regularly follow up with the subjects. Panel studies enable precise collection of personal information, daily activities, addresses, and movement trajectories of patients while requiring relatively low human and material resources. However, statistical validity may be limited due to smaller sample sizes [33]. Cross-sectional studies tend to have larger sample sizes to analyze the effects of long-term exposure and are conducted using different air pollution exposures of populations in different areas. Satellite or monitoring station data are commonly utilized to obtain long-term air pollution levels at a participant’s permanent address, which is then used to determine the patient’s air pollution exposure level [37]. Nevertheless, recording the patient’s movements over the course of several years poses a significant challenge, which may result in exposure evaluations containing inaccuracies. Time-series studies, on the other hand, generally analyze short-term exposures and are conducted using different levels of air pollution exposure in participants at different points in time [34]. Time-series studies are a suitable method for hospitals and community health centers to conduct research, as they collect data over an extended period from one or multiple sites. There are also studies that combine both time and place to calculate participants’ exposure to air pollution [35]. Covariates played a crucial role in examining the relationship between air pollution and lipids. The study included adjusted covariates such as sex, age, body mass index, smoking, alcohol consumption, urban/rural residence, education, economic status, dietary habits, race, medication use, marital status, and physical activity [33,35,39]. These covariates are pertinent to an individual’s health and lipid profile. Additionally, prior research has accounted for the proximity of green space to the address, noise levels [35], and indoor air pollution [37]. These variables have also been examined and linked to changes in lipid profiles [11,40,41]. Studies that consider temporal factors will also adjust for sampling season, temperature, humidity, day of the week of clinical visit, etc. [21,28,34,37]. Several studies have shown that season and temperature may affect lipid profiles [42,43]. Adjusting the day of the week of the clinical visit may be in order to adjust for potential time trends. These covariates were considered relatively comprehensively in the 2023 Raswall et al. study, which demonstrated that increased exposure to PM_2.5_, ultrafine particles (UFP), elemental carbon (EC), and NO_2_ over a period of 30 days was found to elevate non-HDL-C, and higher UFP levels were linked to reduced HDL-C [35]. 

The primary challenge encountered in epidemiological investigations of air pollution’s effects on lipids is the random bias of exposure assessments. People are not constantly outdoors and often travel to various locations, which makes it challenging to precisely measure the air pollution levels that a person is exposed to. Currently, most studies utilize outdoor air pollution levels at the address of the participant to indicate patient exposure, leading to possible air pollution exposure misclassification. This type of exposure misclassification is typically non-differential and can result in an underestimation of the effects of air pollution [37]. Wearable devices provide a means of accurately measuring exposure for small, repeated-measurement studies [44]. Calculating precise levels of air pollution exposure for large sample sizes of study participants remains challenging. Moreover, cross-sectional studies may encounter bias due to regional variations such as confounding influenced by customs, soil, water, and geography. Time-series and repeated-measures studies may also be potentially confounded by time trends, such as simultaneous health awareness improvements and declining air pollution levels. Differences in research methodology and variations in covariates might be the key contributors to the divergent outcomes observed in these studies.

### 2.2. Air Pollutants 

#### 2.2.1. PM_2.5_

The most widely studied air pollutant is PM_2.5_ (particles with aerodynamic diameter less than 2.5 μm), which may be due to PM_2.5_ playing a major toxic role in air pollution, or the easy access of data [6]. The composition of PM_2.5_ is complex, and may include metal particles, inorganic salts, water-soluble ions, black carbon, organic carbon, polycyclic aromatic hydrocarbons, and so on. In addition, its surface may adsorb viruses, bacteria, pollen, endotoxins, and other components [45]. Different components of PM_2.5_ have different effects on blood lipids. The study of He et al. showed that for short-term PM_2.5_ exposure, higher concentrations of tin and lead were significantly associated with the reduction in HDL-C levels, while higher concentrations of nickel were associated with higher HDL-C levels [30]. The study of Li et al. showed that long-term PM_2.5_ exposure was associated with an increased risk of metabolic syndrome in children, and the black carbon component in PM_2.5_ played a major role [27].

#### 2.2.2. Other Air Pollutants

Other air pollutants that are frequently studied include other particulate matter, such as PM_0.1_, PM_1_, PM_10_, and black carbon. It is generally believed that the particles with aerodynamic diameter less than 10 μm (PM_10_) can enter the human body and are mainly deposited in the respiratory tract; PM_2.5_ can enter the alveoli, and PM_0.1_ or UFP can enter human tissues through the respiratory barrier [45]. The gaseous pollutants include SO_2_, NO_2_, CO, ozone (O_3_), etc. SO_2_, NO_2_, and CO mainly come from the combustion of fossil fuels or natural fire and magma. Zhang et al. reported that each 10 μg/m^3^ increment in 3-month average NO_2_ was associated with a 2.59% (95% CI: 1.62–3.57%) increment in LDL-C and 0.85% (95% CI: 0.15–1.55%) increment in HDL-C, while higher SO_2_ levels were associated with higher levels of TC and with lower levels of HDL-C [21]. Li et al. reported that short-term elevated NO_2_, SO_2_, and CO concentrations were associated with decreased HDL-C, apolipoprotein A-I (apoA-I), and decreased cholesterol efflux capacity [28].O_3_ comes from the downward transmission of stratospheric O_3_ and photochemical reactions of nitrogen oxides, volatile organic compounds, and CO under the sunlight. In general, the concentrations of air pollutants are highly correlated, and there is a positive correlation between SO_2_, NO_2_, CO, and particulate matter [7,33,37]. Due to the seasonality of heating and green plants, these pollutants tend to be high in winter and low in summer. However, O_3_ is produced through photochemical reaction, which is serious in summer and light in winter. Therefore, in the time series, the short-term O_3_ concentration is negatively correlated with particulate matter, SO_2_, NO_2_, and CO [7,33]. However, between different regions, the long-term concentration of O_3_ is positively correlated with particulate matter, SO_2_, NO_2_, and CO [37]. With the climate warming and the enlargement of the ozone layer hole, the surface ultraviolet intensity increases. Meanwhile, the increase in the number of motor vehicles leads to an increase in SO_2_ and NO_2_ emissions. In recent years, O_3_ pollution has become more and more serious, gradually attracting higher attention [46,47]. A study that included more than 60,000 people showed that long-term O_3_ exposure was associated with elevated TC and LDL-C and decreased HDL-C [19].

#### 2.2.3. Air Pollutant Mixture

Air pollution exposure is a mixture exposure. It is not clear which components play the main role in the exposure of this mixture and the interaction between them. Because of the high correlation between pollutants and the subsequent multicollinearity problem, considering multiple pollutants at the same time is very difficult and complicated. At present, most studies use single-pollutant models. In single-pollutant model studies, PM_0.1_, PM_1_, PM_2.5_, PM_10_, NO_2_, SO_2_, and O_3_ are all reported to be associated with unhealthy changes in blood lipids [15,19,26,37]. A few studies used the two-pollutant model and the multi-pollutant model. W. Zhang et al. used Bayesian kernel machine regression (BKMR) to analyze the mixed exposure of multiple air pollutants, including PM_2.5_, PM_10_, NO_2_, SO_2_, CO, and O_3_. The results showed that air pollution had adverse effects on HDL-C, LDL-C, non-HDL-C, TC/HDL-C, and non-HDL-C/HDL-C, and O_3_ played an important role in the air pollution mixture [33]. K Zhang et al., using a linear fixed effect model, found that PM_2.5_ played a major role in the two-pollutant model [21]. There are more uncertainties in the analysis of exposure effects of the air pollution mixture. More research is needed to determine the role of various components in the air pollution mixture in order to better evaluate the harm caused by the air pollution mixture.

#### 2.2.4. Indoor Air Pollution

At present, most studies focus on the association between outdoor air pollutants and blood lipids. There is also a small amount of research about indoor air pollution. Sources of indoor air pollution include volatile organic compounds (VOCs) in paint, smoking, cooking, and fireplaces. Wang et al. and Li et al. study household air pollution from cookstoves and they reported that household air pollution (HAP) was associated with elevated human inflammatory markers (such as intercellular adhesion molecule-1, C-reactive protein, and serum amyloid-A) [48] and elevated risk of metabolic syndrome [11]. Xu et al.’s study reported higher levels of TC, LDL-C, and ApoB in restaurant workers exposed to cooking fumes compared to control groups [49]. Dehghani et al. reported that HAP exposure was associated with increased prevalence of high low-density lipoprotein cholesterol, high systolic blood pressure, and high body mass index, and diabetes mellitus-2 in the elderly [50]. However, the effect of indoor air pollution on blood lipids needs more research.

### 2.3. Blood Lipid Indexes 

#### 2.3.1. Introduction to Blood Lipids

The important sources of blood lipids are very low-density lipoprotein (VLDL) particles secreted by the liver and chylomicrons secreted by the small intestine. Because of the instability of TG and cholesterol from food sources, the determination of blood lipids often requires fasting to determine liver-derived lipids [51]. The liver releases TGs, endogenous and exogenous cholesterol into the blood in the form of VLDL particles. Subsequently, VLDL rich in triglycerides and cholesterol undergoes the VLDL1-VLDL2-IDL-LDL delipidation cascade, and then medium-density lipoprotein (IDL) particles and low-density lipoprotein (LDL) particles are generated [51]. Tissue cells endocytose LDL particles into cells via LDL receptor (LDLR). Excess cholesterol can be passively or actively transported to apolipoprotein A-I (ApoA-I) produced by the liver, intestine, and pancreas, forming high-density lipoprotein (HDL) particles. HDL transports cholesterol to steroid organs to produce steroid hormones, or returns to the liver for recycling or clearance [52].

#### 2.3.2. Indicators of Lipid Health

The most common blood lipid indexes are TG, TC, LDL-C, and HDL-C. Epidemiological evidence shows that high levels of LDL-C, TG, and TC are risk factors for cardiovascular disease [4,53,54,55], and high levels of HDL-C are protective factors for cardiovascular disease [55]. Many studies have reported that air pollutants are significantly associated with the increase in TG, TC, and LDL-C or the decrease in HDL-C. However, most studies reported that air pollution was associated with adverse changes in 1–3 lipid indexes, and the changes in the others are beneficial or not significant [6,10,12,13,14,15,16,17,18,20,22,23,24,25,26,27,28,29,30,31,32,33,34,35,38,48]. A few studies reported that air pollution was associated with adverse changes in all four conventional lipid indexes [19,21,56], such as the study of Wang et al., which showed that a 10 μg/m^3^ increase in PM_2.5_ concentration was associated with a 0.92% increase in TC, a 2.23% increase in TG, a 3.04% increase in LDL-C, and a 2.03% decrease in HDL-C [19]. In addition, there are also very few studies that have reported the individual beneficial effects of air pollution, such as the study of Mao et al., in which PM_1_ and PM_2.5_ were associated with the reduction in TG [13,14].

In addition to the common TG, TC, LDL-C, and HDL-C, the outcome indexes also included apolipoprotein A (ApoA), apolipoprotein B (ApoB), the ratio of ApoA to ApoB, low-density lipoprotein particles (LDL-P), oxidized low-density lipoprotein (ox-LDL), non-high-density lipoprotein cholesterol (non-HDL-C), and TC/HDL-C [10,33,56]. ApoA and ApoB are important components of HDL and LDL, respectively. ApoA is highly related to HDL-C levels, while ApoB is highly related to the levels of LDL-C and non-HDL-C [3]. The ratio of ApoB to ApoA-I is more effective in evaluating the risk of coronary artery disease [3]. LDL-P refers to LDL particles, and the concentration of LDL particles is considered to reflect cardiovascular risk better than LDL-C levels [10]. Ox-LDL, the product of LDL oxidation, is an important intermediate in the formation of atherosclerosis [3]. Non-HDL-C (TC minus HDL-C), TC/HDL-C, is a comprehensive index derived from TC and HDL-C. Some studies believe that these comprehensive indexes can better reflect cardiovascular risk [33]. In addition, metabolic syndrome is defined as an outcome according to blood lipid levels and other health indexes. Different studies have slightly different definitions of metabolic syndrome [11,20]. There are also studies that regard dyslipidemia as the outcome. For example, Yan et al. used the definition in *the prevention and treatment guidelines for dyslipidemia in Chinese adults*, in which people with more than one abnormal blood lipid index (TG, TC, LDL-C, or HDL-C) are considered as having dyslipidemia [25]. These studies reflected the harm of air pollution to blood lipids from the perspective of multi-dimensional outcomes. 

### 2.4. Vulnerable Population

Air pollution is widespread in people’s daily life. Studies have found the adverse effects of air pollution on blood lipids in a variety of populations, including healthy people, sick people, urban people, rural people, general adults, middle-aged and elderly people, women, children and adolescents, and some special exposed groups such as pregnant women. According to the Developmental Origins of Health and Disease (DOHaD) theory, adverse experiences during early life may be linked to future illnesses. Mcguinn et al. found that maternal PM_2.5_ exposure in the third trimester of pregnancy was associated with increased TC, LDL, and non-HDL-C and decreased HDL-C in 4-6-year-old children [15].

There are significant differences in the susceptibility of different populations to air pollution. Most studies believe that older people [16,18,21,24], people with a high-fat diet or high energy intake [20,26], and obese people [17,23,24,31,33] are more susceptible to air pollution. Although obesity is detrimental, the study of Kim et al. showed that visceral fat, but not subcutaneous fat, had an interaction with air pollution [17], and the study of Gaio et al. showed that abdominal obesity was associated with the susceptibility to air pollution [23]. There is a close relationship between diabetes and obesity. The study of Yan et al. showed that there was an interaction between fasting blood glucose level and long-term air pollution exposure (PM_2.5_, PM_10_, NO_2_, SO_2_), and the effect of long-term air pollution exposure was more obvious in hyperglycemic people [25]. According to the multiple studies included in the systematic review of Gaio et al., significant results are more likely to be found in the diabetic population than in the normal population [6]. High-fat diet, obesity, and diabetes are also closely related to each other. There is a large intersection between these populations. It is unclear how high-fat diet, obesity, and diabetes affect air pollution susceptibility when acting independently. But what is certain is that people with a high-fat diet, obesity, and diabetes should have health care measures, such as reasonable diet and active exercise. Shin et al.’s study has shown that people who do not exercise are more susceptible [16]. In light of the lack of control we have over our environment, maintaining a healthy weight and engaging in physical activity are effective ways to mitigate the negative effects of air pollution on blood lipid levels. Furthermore, research indicates that utilizing air purifiers indoors can reduce cardiovascular risk factors, providing a potential safeguard for vulnerable individuals exposed to high levels of pollution [57].

Other factors may also impact the vulnerability to air pollution, and these need to be further explored. Some studies have shown that men’s blood lipids were more susceptible to air pollution [14,20], and some studies have shown opposite results [24,31,34]. Regarding sex differences, there is a lack of consistent findings. Li et al.’s study showed that people who do not take drugs are more susceptible [18], but no complete mechanism has been developed to explain why medications decrease air pollution susceptibility, and more research is needed to prove whether medications can prevent the harmful effects of air pollution on blood lipids. Wu et al.’s study showed that there was an interaction of rs505922/rs579459 C allele and short-term PM_10_ exposure in TG, indicating that the effect of air pollution on blood lipids is also influenced by genes [29]. Genetic influences on air pollution susceptibility may help to explain differences in the harms of air pollution in individuals; however, knowledge in this area is still limited. 

## 3. Potential Mechanism of Air Pollution Affecting Lipid Metabolism

### 3.1. Oxidative Stress and Inflammation

**Figure 1:**
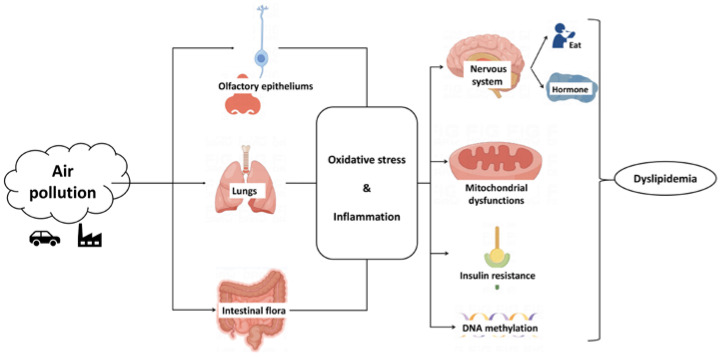
Potential mechanism of air pollution affecting lipid metabolism.

Oxidative stress and inflammation play important roles in the potential molecular mechanism of air pollution affecting blood lipids (Figure 1). Particulate matter can cause oxidative stress and an inflammatory response in the lungs and affect the whole body through cytokines and chemokines [58]. PM_10_ particles can enter the upper respiratory tract, causing allergic and irritating reactions, PM_2.5_ can enter terminal bronchioles and alveoli, and finer particles such as PM_0.1_ can enter the blood, tissues, and other organs through the respiratory barrier. Alveolar macrophages phagocytose PM_2.5_ and release pro-inflammatory mediators, such as IL-12 and IFN-γ, leading to oxidative stress and a systemic inflammatory response [59]. Elements in PM can directly cause lung oxidative damage, and trigger oxidative stress and various damage-related molecular patterns, including oxidized modified lipoprotein, ox-DNA, ssRNA, dsRNA, HMGB1, and mitochondrial proteins, which act on various receptors such as Toll-like receptors and RAGE, and trigger systemic cytokines and chemokines [58]. In addition, PM_2.5_ can also activate NLRP3 inflammasome to induce a systemic inflammatory response, resulting in the elevation of inflammatory markers such as IL-1 α, IL-1 β, IL-6, IL-8, IL-17, IL-18, MIP-3 α, MIP-1 α, MIP-1 β, TNF-α, GM-CSF, and COX-2, which may be related to the endotoxin component adsorbed by PM_2.5_ [59]. In addition to the lungs, particulate matter can also trigger chronic systemic inflammation by inducing nasal and olfactory epithelial damage [60]. Particulate matter ingested through the digestive tract results in inflammation from the digestive tract, such as transforming the intestinal flora into a pro-inflammatory phenotype, destroying the internal integrity of colonic microorganisms, damaging the function of intestinal epithelial cells, and worsening intestinal permeability [61]. Overall, air pollution may cause inflammation through direct contact with the lungs, olfactory nerves, and intestinal flora.

Particle-induced oxidative stress and systemic inflammation can lead to lipid metabolism disorder in adipose tissue. In white adipose tissue, pro-inflammatory mediators (e.g., TNF-α and LPS) can induce mitochondrial dysfunction, which is related to impaired lipolysis and reduced energy consumption (Figure 1). The increase in superoxide anions and the upregulation of Nrf-2 are related to the smaller and fewer mitochondria in white adipose tissue and brown adipose tissue, suggesting the role of oxidative stress [61]. The decrease in energy digestion of adipose tissue may in turn lead to the decrease in the absorption of LDL particles in the blood, affecting the absorption and utilization of LDL-C, TG, and TC in adipose tissue. In addition, the damage of early mitochondrial dysfunction to energy metabolism may lead to a compensatory increase in insulin secretion. Hyperinsulinemia is the early symptom of metabolic disorder [62]. Coincidentally, several studies reported that insulin levels increased in the early stage of air pollution [7,63], which further proves that long-term air pollution exposure may lead to insulin resistance. 

### 3.2. Insulin Resistance

Epidemiological studies have shown that air pollution can increase the risk of insulin resistance [7,64]. Studies have shown that PM_2.5_ may cause insulin resistance in adipose tissue, liver, blood vessels, and other tissues and organs by inhibiting insulin signal transduction [61,65,66]. Cytoregulatory factors JNK, p38 mitogen-activated protein kinase (p38), and extracellular signal-regulated kinase (ERK) can inhibit the insulin signal, while protein kinase B (AKT) mediates insulin signal transduction. Studies have shown that PM_2.5_ exposure is associated with the upregulation of JNK, activation of p 38 and ERK, and inhibition of AKT [61]. Liu et al. found that the activation of p38 and the inhibition of AKT induced by PM_2.5_ are closely related to the CCR2 pathway related to macrophages [67]. Insulin can not only promote the absorption and utilization of blood glucose, but also reduce the secretion of VLDL in the liver [68] and promote the absorption of LDL-C by increasing the expression of the LDL receptor [69]. Therefore, insulin resistance further aggravates the abnormality of LDL-C. (Figure 1)

### 3.3. Hypothalamus

Hypothalamic inflammation also plays an important role in the metabolic dysregulation and insulin resistance induced by particulate matter. Particulate matter exposure can cause inflammation in multiple regions of the brain, including the hypothalamus. There are two possible ways that particulate matter can cause hypothalamic inflammation. One is that particulate matter induces oxidative stress and inflammation in the upper respiratory tract, lungs, and digestive tract, and triggers hypothalamic inflammation through cytokines and chemokines. The other is that particulate matter (especially UFP) directly enters the nervous tissue, increasing the inflammatory response and causing the destruction of the blood–brain barrier [60] In PM_2.5_-exposed mice, Sun et al. found that lipid metabolism disorder was associated with increased expression of kappa B kinase 2, an inhibitor in hypothalamic inflammation [70]. Campolim et al. showed that PM_2.5_ triggered hypothalamic inflammation before obesity. PM_2.5_ exposure impaired leptin signaling in the hypothalamus, resulting in increased food intake and decreased energy expenditure, which may be associated with hypothalamic inflammation [63]. In addition, Li et al.’s randomized double-blind trial showed that short-term PM_2.5_ exposure can activate the hypothalamus–pituitary–adrenal and sympathetic–adrenal–medullary axes, resulting in increased secretion of glucocorticoids, norepinephrine, and epinephrine in healthy adults [57], indicating that PM_2.5_ can change hormone secretion by affecting the central nervous system, thus causing metabolic changes. (Figure 1)

### 3.4. Epigenetic Changes

In addition to oxidative stress and inflammatory response, the mechanism by which air pollution affects blood lipids also includes epigenetic changes. Environmental exposure is considered to have an important link with gene expression modification, and evidence has shown that air pollution may affect DNA methylation. For example, NO_2_ and PM can affect the methylation of the protein kinase C zeta (PRKCZ) gene, which is involved in insulin signaling and is associated with obesity and fasting blood glucose levels. NO_2_ affects the methylation of the ZMIZ1 protein gene, which is related to the changes in blood lipid and blood pressure induced by sex hormones [71]. The study of Li et al. showed that even short-term air pollution exposure can cause changes in DNA methylation [72]. Epigenetics can explain the mechanism of air pollution from another angle. (Figure 1)

## 4. The Potential Diseases Related to Blood Lipid Disorders Caused by Air Pollution

### 4.1. Cardiovascular and Cerebrovascular Diseases

**Figure 2:**
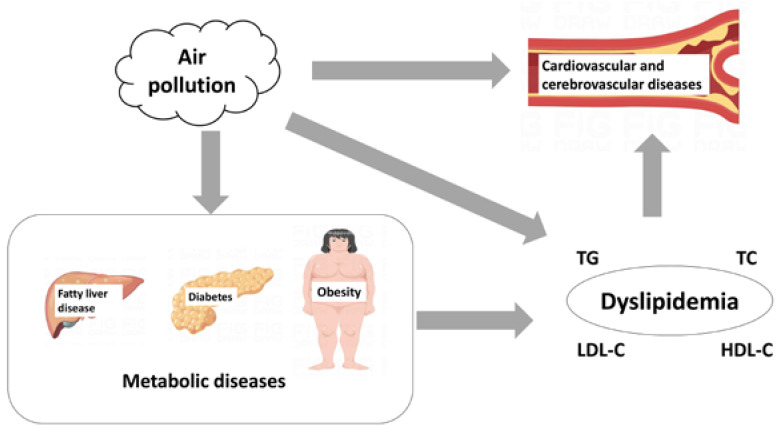
Diseases related to air pollution and blood lipids.

The increased risk of cardiovascular and cerebrovascular diseases is the main harm of dyslipidemia caused by air pollution (Figure 2).

Epidemiological evidence shows that the increase in PM_2.5_ concentration in the short term is associated with an increased risk of cardiovascular events of 1–3%, and long-term PM_2.5_ exposure has a greater impact on the risk of cardiovascular events, up to 10% [73]. According to the scientific statement of the American Heart Association, exposure to PM_2.5_ for a period lasting from a few hours to several weeks can lead to cardiovascular events. Long-term exposure to PM_2.5_ will increase the risk of death from cardiovascular diseases, and the life expectancy of people with high exposure will be shortened by several months to several years. The reduction in PM_2.5_ level is related to the reduction in cardiovascular mortality in just a few years. Many pathological findings provide biological plausibility. Therefore, PM_2.5_ exposure is considered to be a modifiable factor leading to cardiovascular morbidity and mortality [74]. A study on adult patients with cardiovascular disease in China showed that long-term exposure to air pollution was associated with a higher prevalence of cardiometabolic risk factors, and the strongest associations were observed for hyperbetalipoproteinemia, which shows that blood lipids play an important role in air pollution and cardiovascular disease [75].

Long-term and short-term PM_2.5_ is also related to an increase in stroke mortality. A meta-analysis including 103 studies showed that an increase of 10 μg/m^3^ in short-term PM_2.5_ exposure was related to an increase of 1.1% in the hospitalization mortality from stroke [76]. A study conducted in four provinces of China (Sichuan, Shanxi, Guangxi, and Guangdong) showed that the interquartile range (15.14 μg/m^3^) of the annual average PM_2.5_ concentration increase was associated with a 13.7% increase in mortality in stroke patients [77]. A large nationwide cohort study in China showed that for every 10 μg/m^3^ increase in O_3_, the risk of death from total cardiovascular disease increased by 9.3%, the risk of death from ischemic heart disease increased by 18.4%, the risk of stroke increased by 6.3% [78], and the association between ozone and cardiovascular and cerebrovascular diseases did not change after adjusting for particle concentrations [78].

LDL particles are considered as one of the causes of atherosclerosis [3]. LDL particles pass through the arterial wall and enter the extracellular matrix, where they are oxidized. Oxidized LDL induces local inflammation and immune response. Macrophages engulf LDL through the LDLR receptor, form foam cells, and produce cytokines to aggravate the immune cascade reaction, which mobilizes smooth muscle cells to the intima. Smooth muscle cells proliferate and produce an extracellular matrix, forming atherosclerotic plaques [3]. Different from LDL, HDL has the function of reverse cholesterol transport and plays a protective role in the cardiovascular system [79]. It can remove the residual cholesterol from lipoproteins rich in triglycerides and reduce the inflow of cholesterol into the arterial wall [80]. Epidemiological evidence shows that high levels of LDL-C, TG, and TC are risk factors for cardiovascular disease [4,53,54,55], and high levels of HDL-C are protective factors for cardiovascular disease [55]. The meta-analysis by Liu et al. showed that compared with the control group (TG level 90–149 mg/dL), the risk of cardiovascular death increased by 15% (95% CI: 3–29%) and 25% (95% CI: 5–50%) in the mild TG elevation group (150–199 mg/dL) and the high-TG group (≥200 mg/dL), respectively [54]. The meta-analysis of Jung et al. showed that compared with the control, the corresponding HR values for high TC and high LDL-C were 1.27 (95% CI: 1.19–1.36) and 1.21 (95% CI: 1.09–1.35), respectively, while the corresponding HR value of high HDL-C was 0.60 (95% CI: 0.50–0.72) [55]. Similar to cardiovascular disease, high TC, TG, and LDL-C and low HDL-C also lead to a high incidence of cerebrovascular disease [81]. The increase in TG, TC, and LDL-C or the decrease in HDL-C caused by air pollution will promote the formation of atherosclerosis and lead to the occurrence of cardiovascular and cerebrovascular diseases.

### 4.2. Metabolic Dysfunction Diseases

Previous studies have shown that air pollution is associated with metabolic dysfunction diseases, such as fatty liver disease, diabetes, metabolic syndrome, and the increasing prevalence of obesity. All these diseases are associated with dyslipidemia (Figure 2). 

Studies have shown that air pollution is associated with an increased risk of chronic liver disease [82,83,84]. In a cross-sectional study involving 90,086 participants, Guo et al. found that when the 3-year average concentrations of PM_1_, PM_2.5_, PM_10_, and NO_2_ increased by 10 μg/m^3^, the risk of metabolic dysfunction-associated fatty liver disease (MAFLD) increased by 13% (95% CI: 10–17%), 29% (1.25–1.34%), 11% (9–14%), and 15% (12–17%), respectively [82]. A meta-analysis of 16 studies showed that when PM_2.5_ increased by 10 μg/m^3^, the risk of liver cancer, liver cirrhosis, and fatty liver disease increased by 23 (95% CI: 14–33%), 17 (6–29%), and 51% (9–108%), respectively [84]. The increased risk of these diseases suggests that air pollution may lead to abnormal liver lipid metabolism, which has also been confirmed in some animal experiments [85,86,87]. Pathological accumulation of triglycerides and other lipids in hepatocytes is a feature of MAFLD [88]. The balance between synthesis and secretion of triglycerides in the liver is very important for the liver to maintain homeostasis of lipid metabolism. When hepatocyte triglyceride synthesis exceeds VLDL triglyceride secretion, it will lead to the accumulation of triglycerides and further promote hepatic steatosis [88]. In turn, excessive lipid storage in MAFLD will promote the secretion of VLDL, thereby promoting dyslipidemia [88]. MAFLD-related dyslipidemia is characterized by hypertriglyceridemia caused by large VLDL particles (VLDL1), elevated concentrations of small LDL particles, and low HDL-C [89,90]. This kind of dyslipidemia will promote the formation of atherosclerosis. Therefore, atherosclerotic cardiovascular disease is the main cause of death in patients with MAFLD [91].

Type 2 diabetes is also a potential disease that may be caused by air pollution [7,64]. Dyslipidemia of diabetic patients is also characterized by excessive secretion of VLDL in the liver, accompanied by hypertriglyceridemia, increased circulating small dense LDL, and decreased HDL levels [92]. Insulin resistance is one of the reasons for the increase in VLDL secretion in the liver. Physiological fluctuations of insulin regulate VLDL secretion. Increased insulin after a meal inhibits VLDL secretion, while insulin resistance leads to excessive VLDL secretion. The effect of insulin on ApoB synthesis involves the activation of phosphoinositide 3-kinase (PI 3-K), which enables the production of downstream phosphatidylinositol (4,5,3) triphosphate and inhibits VLDL production [93,94]. In addition, insulin resistance also involves Foxa2 and PGC-1 β synergistically, inducing the expression of MTTP, thereby increasing VLDL secretion [95]. Therefore, air pollution may also lead to insulin resistance and then induce dyslipidemia.

Air pollution exposure increases the risk of obesity in both adults and children and adolescents. A meta-analysis of 15 studies showed that among children and adolescents, PM1, PM_2.5_, PM_10_, and NO_2_ (before a 10 μg/m^3^ increase) were associated with 41%, 28%, 12%, and 11% increased risk of obesity, respectively [96]. A meta-analysis revealed that among adults, elevated NO_2_, SO_2_, and O_3_ increases the risk of obesity by 13%, 4%, and 7%, respectively [97]. Obesity often leads to an increase in blood lipid levels [98,99]. For example, the study of Guo et al. showed that compared with normal weight subjects, overweight and obese subjects had significantly higher LDL-C and lower HDL-C, while abdominal obesity was associated with higher TG after multiple-factor adjustment [99]. The cause of obesity may be related to the metabolic dysfunction of white adipose tissue and brown adipose tissue. For example, oxidative stress and inflammation caused by PM_2.5_ will damage mitochondrial function and inhibit oxygen consumption and lipid oxidation. These changes will lead to the whitening of brown adipocytes and stimulate the storage of TG in white adipocytes and the hypertrophy of adipocytes [61,65].

A variety of metabolic diseases related to air pollution are related to dyslipidemia, which makes it biologically reasonable that air pollution leads to dyslipidemia. Fatty liver disease mainly reflects the abnormality of lipid metabolism in the liver. Insulin in diabetes affects all parts of the body, including the liver, skeletal muscle, and adipose tissue. Obesity is mainly reflected in the adipose tissue of the body. Under the complex influence of air pollution, these tissues and organs jointly lead to dyslipidemia.

## 5. Summary and Prospect

Most of the evidence from epidemiological studies suggests that air pollution has a detrimental impact on blood lipids. In addition, this phenomenon is substantiated by mechanistic studies. Air pollution may affect blood lipids through oxidative stress, inflammation, insulin resistance, mitochondrial dysfunction, hypothalamic hormones, and epigenetic changes. Metabolic diseases related to air pollution, such as fatty liver disease, diabetes, and obesity, are associated with dyslipidemia. The overall study supports that air pollution affects blood lipids. Among different groups of people, the elderly and people with a high-fat diet, obesity, and diabetes need to prevent the harm caused by air pollution. 

However, additional research is required to clarify the health impacts of indoor air pollution on blood lipids, the causal mechanisms behind the impact of gaseous pollutants such as NO_2_, SO_2_, CO, and O_3_ on blood lipids, the factors influencing differences in air pollution vulnerability among populations, and effective strategies for reducing risks associated with air pollution exposure. Moreover, improved methodologies may be necessary for assessing the impact of mixed air pollution. 

Literature search methods: The research has been carried out on Web of Science and Pubmed. The search strategy was assessed by alternatively combining the keywords “lipid”, “dyslipidemia” with “PM”, “particulate matter”, “NO_2_”, “SO_2_”, “O_3_”, “CO”, and “air pollution”.

## Data Availability

Not applicable.

## Abbreviations

LDL-C: low-density lipoprotein cholesterol; HDL-C, high-density lipoprotein cholesterol; TG, triglyceride; TC, total cholesterol; FBG, fasting blood glucose; PM_2.5_, particulate matter 2.5; PM_10_, particulate matter 10; NO_2_, nitrogen dioxide; SO_2_, sulfur dioxide; CO, carbon monoxide; O_3_, ozone; GBD, Global Burden of Disease, Injuries, and Risk Factors; DALY, disability adjusted life year; VLDL, very low-density lipoprotein; IDL, medium-density lipoprotein; ApoA, apolipoprotein A; ApoB, apolipoprotein A; LDL-P, low-density lipoprotein particles; ox-LDL, oxidized low-density lipoprotein; HAP, household air pollution; non-HDL-C, non-high-density lipoprotein cholesterol; DOHaD, Developmental Origins of Health and Disease; VOCs, volatile organic compounds; LDLR, LDL receptor; MAFLD, metabolic dysfunction-associated fatty liver disease; TNF, tumor necrosis factor; IL, interleukin; JNK, C-Jun N-terminal kinase; PI 3-K, phosphoinositide 3-kinase; ERK, extracellular signal-regulated kinase; AKT, Protein kinase B; CCR-2, C-C chemokine receptor type 2.
